# Prevention of advanced glycation end-products formation in diabetic rats through beta-cell modulation by *Aegle marmelos*

**DOI:** 10.1186/s12906-017-1743-y

**Published:** 2017-04-21

**Authors:** Rahman M. Hafizur, Shahrukh Momin, Noor Fatima

**Affiliations:** 0000 0001 0219 3705 grid.266518.e Dr. Panjwani Center for Molecular Medicine and Drug Research, International Center for Chemical and Biological Sciences (ICCBS), University of Karachi, Karachi, 75270 Pakistan

**Keywords:** *Aegle marmelos*, Advanced glycation end-products, Immunohistochemistry, β-cell function, Insulin secretion, Eugenol

## Abstract

**Background:**

Although the anti-diabetic activity of *Aegle marmelos* (AM) is known, however, its anti-glycation activity is not reported yet. In this study, we have investigated its anti-glycation activity under in vitro and in vivo conditions and determined possible mechanism(s) in streptozotocin-induced diabetic rats.

**Methods:**

Effective dose of AM (400 mg/kg) was administrated orally to diabetic rats for 42 days. Thereafter, blood glucose, serum insulin, HbA1c, antioxidant status, and advanced glycation end-products (AGEs) were measured. AGEs and its receptor (RAGE) in kidney were analyzed by immunohistochemistry and immunoblotting. Additionally, pancreatic sections were co-stained for insulin and glucagon and images were acquired using NIKON TE2000E fluorescence microscopy.

**Results:**

Oral administration of AM extract resulted in a significant increase in serum insulin by better functioning of β-cell and preserving pancreatic β-cell integrity in diabetic rats. Treatment of AM extract significantly (*p* = 0.000) prevented the formation of HbA1c in the diabetic rats (8.20 ± 0.18% *vs*. 11.92 ± 0.59%). The circulatory AGEs level found in diabetic rat was significantly (*p* = 0.002) attenuated by AM treatment (0.66 ± 0.05 mg/ml *vs*. 1.18 ± 0.19 mg/ml). AM treatment also reduced AGEs accumulation around Bowman’s capsule and in tubular basement membrane around arteries in diabetic rat kidney. The accumulation of RAGE was very similar to that of AGEs in diabetic rats and RAGE accumulation was also prevented by AM treatment. The extract showed potent antioxidant activity both under in vitro and in vivo systems. Eugenol, one of the active constituent of AM fruit extract, showed acute blood glucose-lowering activity in diabetic rats and enhanced glucose-stimulated insulin secretion from mice islets.

**Conclusion:**

AM extract prevents AGEs formation by modulating β-cell function, and eugenol may play important role in preventing complications of diabetes in this rat model.

## Background

Advanced glycation end products (AGEs) are complex and heterogeneous group of compounds that are generated through a non-enzymatic glycation and oxidation of proteins, lipids and nucleic acids. These AGEs accumulate and stimulate diabetic complications in diabetic subjects. It involves the reaction of amino group of proteins, lipids and nucleic acids with carbonyl group of reducing sugars to form a reversible Schiff base. The Schiff base rearranges to form Amadori products that are irreversible. Over the course of days to weeks, these Amadori products undergo further rearrangement reactions to form the irreversibly bound moieties known as AGEs [1]. AGE-modified proteins may be part of the mechanism leading to development and progression of diabetic complications. The best known example of an Amadori product is the glycated hemoglobin (HbA1c). Protein glycation in diabetic patients increases up to 10-folds than normal subjects [2].

Many synthetic compounds have been evaluated as inhibitors of the AGEs formation, but none have yet been approved for clinical use. Aminoguanidine, an antiglycating agent reached upto phase III clinical trial, but due to various side effects it was discontinued [3]. Several other AGEs inhibitors and cross-link breakers including GLY-230, TRC4186, TRC4149, pyridoxamine, ALT-711, ALT-946, OPB-9195, LR-90, LR-74, LR-9 and N-phenacylthiazolium bromide are under various clinical phases [4]. Considering these complications, development of drugs for prevention of AGEs formation for commercial use takes considerable duration. Therefore, alternative therapy such as dietary compounds with anti-glycating activities can be good alternative agents for the prevention of AGEs.

*Aegle marmelos* (AM), commonly known as bael, is a spinous tree belongs to the family Rutaceae. It is widely found in India, Bangladesh, Myanmar and Sri Lanka. AM has an important place in indigenous system of medicine. Its fruit, leaves, root, bark, and flowers are highly valued in Ayurvedic medicine in India. In fact, as per Charaka (1500 BC) no drug has been longer or better known or appreciated by the inhabitants of India than the AM. The AM fruit pulp contains many functional and bioactive compounds such as carotenoids, phenols, alkaloids, coumarins, flavonoids, and terpenoids. In addition, it also contains many vitamins and minerals including vitamin C, vitamin A, thiamine, riboflavin, niacin, calcium, and phosphorus [5, 6]. In addition, more than 100 compounds have been isolated from AM fruit juice [7–9]. Due to the presence of these bioactive compounds, AM is found to be one of the important dietary adjuncts used in the indigenous traditional medicine.

Pharmacological studies have validated most of the ethno-medicinal uses of AM, including its use as an anti-diabetic, anti-microbial, anti-microfilarial, anti-fungal, hypoglycemic, astringent, anti-diarrheal, anti-dysentric, demulcent, analgesic, anti-inflammatory, anti-pyretic, anti-dyslipidemic, immunomodulatory, anti-proliferative, wound-healing, insecticidal, anti-cancer, and cardioprotective agent [10].

Different parts of AM plant (fruit, leaves, root, bark and flowers) have reported anti-diabetic activity [11, 12]. In another study, it was found that AM significantly decreased the HbA1c in diabetic rats [13]. However, the preventive role of AM on AGEs formation and mechanism(s) are not yet known. The present study was designed to elucidate the anti-glycation activity of AM and to explore possible mechanism(s) in streptozotocin-induced diabetic rats.

## Methods

### Materials

Streptozotocin (STZ), glibenclamide, tolbutamide, collagenase-V, bovine serum albumin (BSA), and aminoguanidine, and eugenol (99% pure) were obtained from Sigma (St. Louis, MO, USA). Rat HbA1c, creatinine and rat and mouse insulin ELISA kits from Crystal Chem Inc. (Downers Grove, USA). Triglycerides, total cholesterol, HDL-cholesterol and total antioxidant status assay kits from Randox (Crumlin, UK). Rat and mouse ultra-sensitive insulin ELISA kits were from Crystal Chem Inc. (IL, USA). Rat AGEs ELISA kit was purchased from Cell Biolabs, Inc. (San Diego, CA, USA). Alanine aminotransferase (ALT), aspartate aminotransferase (AST) and urea test strips were purchased from Roche Diagnostics (Mannheim, Germany). Primary antibodies for insulin (guinea-pig polyclonal to insulin), AGEs (rabbit polyclonal to AGEs), CML (mouse monoclonal to CML), and RAGE (rabbit polyclonal to RAGE) were obtained from Abcam (Cambridge, UK), and glucagon (Clone K79bB10) and β-actin (A1978) from Sigma. The secondary antibodies, Texas Red-donkey anti-guinea-pig IgG, Cy2-donkey anti-mouse IgG peroxidase-conjugated goat anti-rabbit IgG and peroxidase-conjugated goat anti-mouse IgG were purchased from Jackson Immunoresearch (Baltimore, PA, USA), and Alexa-647-goat anti-rabbit IgG and Alexa-594-goat anti-rabbit IgG from Invitrogen (Eugene, Oregon, USA). 4′,6-Diamidino-2-phenylindole (DAPI) was obtained from Wako Pure Chemical (Osaka, Japan).

### Preparation of AM extracts

Ripe fruit of AM (5.0 kg) was purchased from a local market of Karachi, Pakistan, by single supplier, and verified from Herbarium of Botany Department of Karachi University, Pakistan (No. 86469). The pulp (4.6 kg) was collected and seeds were removed and soaked into aqueous 80% methanol (3 × 4 L) for 72 h each time at room temperature. Pooled extracts were filtered, combined and evaporated to dryness under vacuum by using rotary evaporator. Finally, the crude extract was freeze dried to give the experimental extract (157.5 g). The percentage yield of the extract was 3.57%. The obtained extract was kept at 4 °C until used. The extract was dissolved in distilled water before administration to rats.

### Toxicity evaluation

The extract of AM was evaluated for toxicity in rats. To determine acute toxicity, single oral administration of AM extract at different doses (500, 1000, and 2000 mg/kg body weight) was done to different groups of Wistar rats (*n* = 6) of either sex. Mortality and general behavior of the animals were observed continuously for the initial 4 h and intermittently for the next 6 h and then again at 24, 48 and 72 h following extract administration. To determine chronic toxicity autopsy was performed after 42 days of extract treatment to check any abnormality in the liver, kidney, gastrointestinal tract, spleen and heart. Serum creatinine and urea, ALT and AST were also evaluated to check the toxicity to kidney and liver, respectively. Eugenol (EG) was evaluated for its cytotoxicity in pancreatic MIN6 cells, and no toxic effects were observed (IC_50_ > 200 μM).

### Animals and induction of diabetes

Wistar rats (180–250 g) of both sexes from Animal House of International Center for Chemical and Biological Sciences (ICCBS), University of Karachi, were used throughout the experiment after ethical clearance from the Animal Use Committee of the ICCBS (Animal study protocol number: 2015–0020). The animals were housed in clean cages under a constant 12-h light-dark cycle with free access to water and food. A 55 mg/kg solution of STZ in citrate buffer (0.1 M, pH 4.5) was intraperitoneally injected to overnight fasted rats. Non-diabetic control rats were injected with citrate buffer (vehicle, pH 4.5) only. After 7 days of STZ induction, fasting blood glucose levels were determined using a glucometer. Rats having fasting blood glucose above 15 mM were selected for the study.

### Oral glucose tolerance test (OGTT) with AM extract in diabetic rats

Diabetic rats (*n* = 30) were divided into five groups (6 rats/group). Group 1, diabetic rats was received only distilled water (Db); Group 2–4, diabetic rats was received AM extract of 200 (AM 200), 400 (AM 400), and 600 (AM 600) mg/kg, respectively; Group 5, diabetic rats was received 5 mg/kg glibenclamide (Gb). All the diabetic rats were fasted overnight (14-h) prior to the OGTT test. Sixty mins following the AM extract or Gb administration, 3 g/kg oral glucose load was given to each rat. Blood glucose levels were measured 60 min before the administration of AM and at 0, 30, 60, 120, and 180 min after glucose load.

### Chronic extract treatment

The experimental rats were divided into five groups. Group I: non-diabetic control rats; Group II: untreated diabetic rats; Group III: AM*-*treated diabetic rats; Group IV: Gb*-*treated diabetic rats; Group V: aminoguanidine*-*treated diabetic rats. In the case of AM-treated diabetic rats the extract was fed orally at a dose of 400 mg/kg once daily for 42 days. Non-diabetic control rats and untreated diabetic rats received equal volume of water in place of the extract, while Gb (5 mg/kg) was given orally to the Gb*-*treated diabetic rats. AG was given in drinking water (1 g/l, freshly prepared) daily to the AG*-*treated diabetic rats.

### Collection of blood samples and estimation of biochemical parameters

For biochemical estimation, blood samples were collected from the tail vein on day 1 after overnight fasting. Finally after 42 days of extract treatment, overnight fasted rats were sacrificed and blood was collected for biochemical parameters. HbA1c was measured from EDTA blood by rat HbA1c assay kit. Remaining blood samples were allowed to clot and serum was separated by centrifugation. Serum samples were stored at −40 °C for biochemical assays.

Estimation of serum insulin and serum AGEs were performed by ELISA. Serum triglyceride was measured by enzymatic colorimetric method and total cholesterol and HDL-cholesterol were measured by cholesterol oxidase/peroxidase method. Total antioxidant status was measured using ABTS® substrate assay kit. Serum creatinine was measured by a rat creatinine assay kit according to the manufacturer’s protocol. Serum ALT and AST were measured by standard techniques using the Reflotron® Plus Dry Chemistry Analyzer.

### Assessment of β-cell function and insulinogenic index

β-cell function (HOMA-B%) was measured from fasting glucose (mM) and fasting serum insulin (pM) concentration by homeostasis model assessment (HOMA) using HOMA-CIGMA software, where HOMA-B% represents β-cell function [14]. Insulinogenic index (IGI), a frequently used index of β-cell function, was calculated as insulin (pM) /glucose (mM).

### Hematoxylin and eosin staining

For hematoxylin and eosin (H&E) staining, pancreatic sections were deparaffinized in xylene, rehydrated in graded 2-propanol series and washed in water. The sections were then stained with H&E. Pancreatic sections were viewed using a Nikon 90*i* microscope (Nikon, Tokyo, Japan) and the images were acquired with a Nikon DXM 1200C camera using NIS-Elements image analysis software AR 3.0 (Nikon).

### Periodic acid-Schiff (PAS) staining

For PAS staining, slides of kidney sections were selected from all experimental groups. After deparffinization and re-hydration, the slides were washed with deionized water for 10 min. One percent periodic acid solution was added to sections with dropper and kept for 10 min. After that the slides were rinsed with running tap water for 10 min and then rinsed with deionized water for 2 min, two times. Schiff’s reagent was added to sections with dropper and left for 5–7 min. The slides were rinsed with warm (42 °C) water for 5 min and then rinsed quickly with deionized water. The slides were stained with Mayer’s Hemalaum solution for 3 min and then rinsed with running tap water for 7 min. The slides were washed, dehydrated, cleared in xylene and finally mounted with mounting media using clean cover slips. Sections were viewed using a Nikon 90*i* microscope and the images were acquired with a Nikon DXM 1200C camera using NIS-Elements image analysis software AR 3.0. Finally, image processing was performed with Adobe Photoshop CS2.

### Immunohistochemistry

Insulin and glucagon immunostaining was performed as described previously [15, 16]. For AGEs and RAGE immunostaining, kidney sections from all experimental groups were deparaffinised, rehydrated, washed in water and subjected to antigen retrieval (90 °C for 30 min) in 0.1 M citrate buffer (pH 6.0). After blocking each selected section was incubated with AGEs (1:100) or RAGE antibody (1:100) for 1 h. After washing with PBS, sections were incubated with Alexa 647-goat anti-rabbit IgG (1:100) or Alexa 594-goat anti-rabbit IgG (1:100) for 45 min. Finally, the nuclei were stained with DAPI, washed with PBS and mounted in Fluoromount solution. The fluorescent images were visualized using a Nikon TE2000E fluorescent microscope equipped with a Nikon DS-2MBWc camera in DAPI, Cy3 and Cy5 channels. The images were acquired using NIS-Elements image analysis software AR 3.0. and image processing was performed with Adobe Photoshop CS2. As negative control primary antibodies were excluded and no specific immunostaining was observed.

### Western blot analysis

Kidney tissues were lysed in RIPA buffer (50 mM TrisHCl, pH 7.5, 150 mM NaCl, 1% Triton X-100, 0.1% SDS, 1 mM EDTA, 5 mM β-glycerophosphate, 1 mM Na_3_VO_4_, 1 mM NaF) containing 1× complete protease inhibitor cocktail on ice. Aliquots containing 50 μg proteins were resolved using SDS-10% PAGE (Bio-Rad Mini-PROTEAN Precast Gels) and transferred onto nitrocellulose membranes by electroblotting using a semidry transfer apparatus (Bio-Rad Laboratories, Hercules, CA, USA). Membranes were incubated overnight at room temperature under agitation with Tris-Buffered Saline(TBS)-0.5% Tween 20 (TBST) plus 5% nonfat milk (NFM) to block the non-specific reactivity, and they were then probed for 2 h at room temperature with primary antibody diluted 1:1000 anti-AGEs, 1:1000 anti-RAGE, and 1:1000 anti-CML in TBST plus 1% NFM. Subsequently, membranes were washed and incubated for 1 h at room temperature with either peroxidase-conjugated goat anti-rabbit IgG or peroxidase-conjugated goat anti-mouse IgG diluted 1:2000 in TBST plus 1% NFM. Finally, membranes were washed and developed with an enhanced chemiluminescence Western blotting reagent (GE Healthcare Life Sciences, UK).

### Acute eugenol treatment

Eugenol (EG), one of the active constituent of AM fruit extract, was fed at a dose of 50 mg/kg to overnight fasted non-diabetic and diabetic rats, and blood glucose levels were measured at 0, 60, and 120 min. Non-diabetic and diabetic control rats were received 2 mL of water in place of EG.

Isolation of islets from mouse and insulin secretion assay.

Isolation of islets and insulin secretion assay was performed as described previously [17, 18]. In brief, islets (*n* = 3) were incubated for 60 min in KRB buffer solution with 3 mM (basal) or 16.7 mM (stimulatory) glucose supplemented with EG in different concentrations. After incubation, 100 μL aliquots were taken from each tube and insulin in the incubation buffer was measured using mouse insulin ELISA kit.

### Statistical analysis

All statistical analyses were performed by using the SPSS (Statistical Package for Social Science) package for Windows version 12.0 (SPSS, Inc., Chicago, IL, USA). All values are expressed as mean ± SEM. To compare data between and within group, unpaired and paired *t*-tests (2-tailed) were performed. One-way ANOVA followed by post hoc Dunnett’s test was used to compare the differences among the groups. *P*-values less than 0.05 (*p* < 0.05) were considered as statistically significant.

## Results

### Toxicity evaluation of AM extract

The acute toxicity results showed no mortality up to the dose of 2000 mg/kg body weight. Serum creatinine, urea, ALT and AST were measured as a marker of toxicity to check whether the AM extract have any toxic effect on chronic (42 days) administration. The results showed that there was no significant increase in the serum creatinine, urea, ALT and AST levels during the experimental period.

### Effects of AM extract on OGTT in diabetic rats

**Fig. 1:**
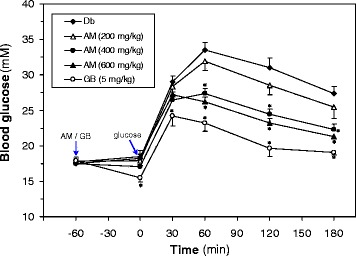
Effects of different doses of *A. marmelos* extract on OGTT in STZ-induced diabetic rats. *A. marmelos* extract was administered orally 60 min before glucose (3 g/kg body weight) loading. Blood was sampled from the tail vein at −60 (before *A. marmelos* extracts and glibenclamide) and at 0, 30, 60, 120, and 180 min after glucose load. Data represent mean ± SEM for 6–9 rats in each group. Db, diabetic rats treated with glucose only; GB, diabetic rats treated with glucose plus glibenclamide (5 mg/kg); AM 200, AM 400 and AM 600, diabetic rats treated with glucose plus *A. marmelos* extract of 200, 400, 600 mg/kg, respectively. *p < 0.05 compared with the corresponding values of vehicle group of Db rats (two-way ANOVA with Dunnett’s multicomparison test)

First, we examined the effects of oral administration of three different doses of AM extract on blood glucose levels of STZ-induced diabetic rats challenged with a glucose load (Fig. 1). We found that AM extract improved glucose tolerance in a dose-dependent manner. The blood glucose level of the AM group showed a tendency to decrease at 60, 120 and 180 min, as compared with the vehicle. At 60 min, the 400 mg/kg and 600 mg/kg doses of the AM extract significantly (F(4,28) = 2.71, *p* = 0.04) lowered blood glucose levels compared to the vehicle. A decrease of 18.5% (*p* = 0.04) was observed with 400 mg/kg of extract at 60 min. The dose of 600 mg/kg also produced a significant (*p* = 0.04) decrease (21.8%) in the blood glucose at 60 min. However, the dose of 200 mg/kg did not produce a significant decrease (4.6%) in the blood glucose at 60 min. AM 400 mg/kg also improved glucose at 120 and 180 min, respectively. Similar results were also found in AM 600 mg/kg group. The standard drug, glibenclamide (5 mg/kg), caused significant decrease in the blood glucose levels at 30, 60, 120 and 180 mins, which was comparable to the doses of 400 mg/kg and 600 mg/kg of AM, respectively.

### Effect of chronic *A. marmelos* extract treatment on body weight and fasting blood glucose in diabetic rats

**Fig. 2:**
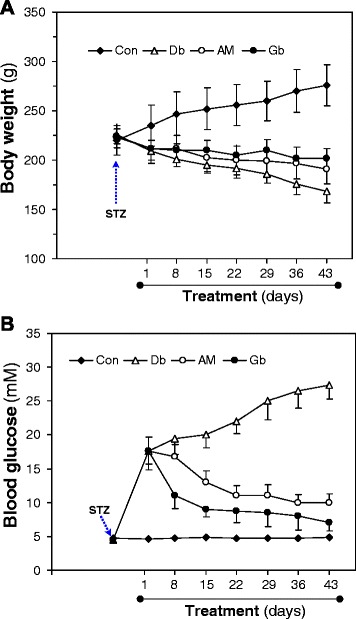
Effect of chronic *A. marmelos* extract treatment on body weight and fasting blood glucose in diabetic rats. The changes of body weight (**a)** and fasting blood glucose (**b)** were evaluated every week during the treatment periods. Data represent mean ± SEM for 6–11 rats in each group. Control, non-diabetic control rats; Db, untreated diabetic rats; AM, *A. marmelos*-treated diabetic rats; Gb, glibenclamide-treated diabetic rats. *p < 0.05, **p < 0.01, ***p < 0.001 compared with Db rats (one-way ANOVA with Dunnett’s multicomparison test)

Next, we examined the effect of AM treatment on the variations in body weight and fasting blood glucose of normal control, untreated diabetic and diabetic rats (Fig. 2). During the experimental period, we observed a gradual gain in body weight in the control rats in contrast to a gradual reduction in body weight in the STZ-induced rats (Fig. 2a). A significant (*t* = 6.3, *p <* 0.001) decrease in body weights was observed after 15 days of STZ-induction compared to their initial day value. More significant (*t* = 10.14, *p <* =0.001) decrease in body weights was observed from day 22–43 of STZ-induction. In contrast, when the diabetic rats were treated with AM (400 mg/kg) extract or Gb (5 mg/kg) for 42 days, their body weights were improved significantly (F(3,8) = 26.45, *p* < 0.001) (Fig. 2a).

After 7 days of STZ-induction, there was a sharp increase in fasting blood glucose in the STZ-induced rats (17.57 ± 2.08 mM vs. 4.48 ± 0.49 mM; *t* = −4.47, *p* = 0.004) (Fig. 2b). From day 7 to onwards, blood glucose increased gradually but slowly in the untreated diabetic rats. Interestingly, when the diabetic rats were treated with AM extract at a dose of 400 mg/kg/day for 42 days, the extract reduced the elevated blood glucose significantly (day 1, 17.38 ± 0.36 mM; day 43, 9.97 ± 1.02 mM; F(3,26) = 55.072, *p* < 0.001). The Gb also reduced the fasting blood glucose (7.08 ± 0.29 mM) significantly (*p* < 0.001). On the other hand, very little changes were found in the fasting blood glucose of control rats during the experimental period.

### Effect of chronic AM extract treatment on fasting serum insulin, insulinogenic index and β-cell function in diabetic rats

We investigated the changes of fasting serum insulin and β-cell function in diabetic rats as shown in Table 1. The fasting serum insulin level was decreased dramatically (F(4,32) = 33.48, *p* < 0.001) in the diabetic rats (34.4 ± 1.72 pM) compared to control rats (170.3 ± 22.3 pM). In the untreated diabetic rats, the fasting serum insulin also decreased significantly during the experimental period (day 1, 34.4 ± 1.72 pM; day 43, 27.5 ± 3.4 pM; *t* = 3.42, *p* = 0.011). After 42 days of extract treatment, a significant (*p* < 0.001) increase of serum insulin was found in AM-treated diabetic rats (106.6 ± 12.0 pM) compared to the untreated diabetic rats (27.5 ± 3.4 pM). Gb also increased serum insulin level (87.7 ± 10.3 pM) significantly (*p* = 0.011).

**Table 1:** Effect of chronic *Aegle marmelos* fruit extract on insulinemic status in diabetic rats

Groups	Fasting serum insulin (pM)		Insulinogenic index (pM insulin/mM glucose)		β-cell function (%)	
	Day 1	Day 43	Day 1	Day 43	Day 1	Day 43
Control (*n* = 11)	170.3 ± 22.3	172.1 ± 27.5	37.21 ± 4.55	36.71 ± 3.72	136.25 ± 16.63	142.02 ± 21.38
Db (*n* = 8)	34.4 ± 1.72	27.5 ± 3.4	1.95 ± 1.13	1.60 ± 0.47	20.17 ± 3.57	11.54 ± 1.56
AM (*n* = 6)	39.5 ± 6.8	106.7 ± 12.0**	2.27 ± 0.62	10.76 ± 1.21**	21.07 ± 2.83	68.32 ± 4.56**
Gb (*n* = 6)	32.6 ± 6.8	87.7 ± 10.3*	2.17 ± 0.89	12.32 ± 1.06**	21.41 ± 3.11	74.85 ± 6.07**

All values were expressed as mean ± SEM Control, non-diabetic control rats; Db, untreated diabetic rats; AM, *Aegle marmelos-*treated rats*;* Gb, glibenclamide-treated rats. Insulinogenic index: Insulin (pM) / glucose (mM). *n* = number of rats in each group. *p < 0.01, **p < 0.001, compared with Db values (one-way ANOVA with Dunnett’s multicomparison test)

Furthermore, we calculated the insulin secretion by insulinogenic index (IGI) and it was found that IGI was 10-fold higher (F(1,12) = 11.64, *p* = 0.005) in AM-treated diabetic rats (10.7 ± 1. pM insulin/mM glucose) compared to the untreated diabetic rats (1.00 ± 0.4 pM insulin/mM glucose). The IGI data clearly suggest that AM treatment improved insulin secretion in diabetic rats.

In control rats, β-cell function was found 136.25 ± 16.63% on day 1 (Table 1). No significant change in β-cell function was observed during the experimental period (day 43, 142.02 ± 21.38%). In diabetic rats, β-cell function was dramatically decreased compared to the control rats (20.17 ± 3.57% *vs.* 136.25 ± 16.63%; F(4,32) = 39.22, *p* < 0.001). Almost 80% β-cell destruction was observed in this diabetic model rats. In the untreated diabetic rats, β-cell function was decreased very close to the significant level during the experimental period (day 1, 20.17 ± 3.57%; day 43, 11.54 ± 1.56%; *p* = 0.057). Interestingly, after 42 days of extract treatment, β-cell function was increased significantly in AM-treated group (68.32 ± 4.56%, *p* < 0.001) compared to the untreated diabetic group (11.54 ± 1.56%). The Gb also improved β-cell function significantly (*p* < 0.001).

### Effect of chronic AM extract treatment on total cholesterol, triglycerides, HDL-cholesterol and LDL-cholesterol levels

Table 2 shows the chronic effect of AM extract on total cholesterol, triglycerides, HDL- and LDL-cholesterol levels. STZ-induced diabetic rats did not show a significant change in the total serum cholesterol during the experimental periods. Even the administration of AM or Gb did not significantly affect the total cholesterol level.

**Table 2:** Effect of chronic *A. marmelos* extract treatment on lipid profiles in diabetic rats

Groups	Total cholesterol (mg/dL)		Triglycerides (mg/dL)		HDL-cholesterol (mg/dL)		LDL-cholesterol (mg/dL)	
	Day 1	Day 43	Day 1	Day 43	Day 1	Day 43	Day 1	Day 43
Control (*n* = 11)	47.3 ± 2.4	49.9 ± 2.4	33.8 ± 2.5	35.4 ± 2.4	26.8 ± 1.4	25.7 ± 1.3	18.2 ± 3.3	19.2 ± 2.86
Db (*n* = 8)	45.3 ± 2.4	40.1 ± 1.7	49.9 ± 3.5	54.2 ± 3.1	12.0 ± 1.0	10.3 ± 1.2	23.1 ± 2.6	26.9 ± 1.92
AM (*n* = 6)	44.6 ± 1.2	43.0 ± 3.0	50.6 ± 3.1	36.1 ± 3.5***	13.3 ± 1.0	20.1 ± 1.3***	21.7 ± 1.8	15.6 ± 2.20*
Gb (*n* = 6)	42.8 ± 2.8	46.0 ± 3.2	50.5 ± 2.8	39.0 ± 1.1**	14.1 ± 1.7	18.0 ± 1.1**	22.7 ± 1.6	20.1 ± 3.11

All values were expressed as mean ± SEM. Control, non-diabetic control rats; Db, untreated diabetic rats; AM, *A. marmelos-*treated rats*;* Gb, glibenclamide-treated rats. *n* = number of rats in each group. *p < 0.05, **p < 0.01, ***p < 0.001, compared with Db values (one-way ANOVA with Dunnett’s multicomparison test)

In the diabetic rats, triglycerides level was significantly (F(4,32) = 9.556, *p <* 0.001) increased (54.2 ± 3.1 mg/dl) compared to the control rats (35.5 ± 2.4 mg/dl) at the end of the experimental period. After 42 days of AM extract treatment, triglycerides level was significantly (*p* < 0.001) decreased in AM-treated group (36.1 ± 3.5 mg/dl). Gb also reduced triglycerides levels significantly (39.0 ± 1.1 mg/dl, *p* = 0.001).

The HDL-cholesterol level decreased significantly in the diabetic rats compared to control rats (12.0 ± 1.0 mg/dl *vs.* 26.8 ± 1.4 mg/dl; F(4,32) = 16.054, *p <* 0.001). After 42 days of AM extract treatment, HDL-cholesterol level was increased significantly in AM-treated group (20.1 ± 1.3 mg/dl, *p* < 0.001) compared to the untreated diabetic group (10.3 ± 1.2 mg/dl). Gb also increased HDL-cholesterol level significantly (18.0 ± 1.2 mg/dl, *p =* 0.001).

In the diabetic rats, the LDL-cholesterol level was slightly increased compared to the control group (23.1 ± 2.6 mg/dl *vs.* 18.2 ± 3.3 mg/dl). However, after 42 days treatment of AM extract, LDL-cholesterol levels were reduced significantly in AM-treated group (15.6 ± 2.2 mg/dl; F(4,32) = 3.517, *p =* 0.017) compared to the untreated diabetic group (26.9 ± 1.9 mg/dl). Gb did not change the LDL-cholesterol levels significantly (20.2 ± 3.1 mg/dl).

### Effect of chronic AM extract treatment on HbA1c level

The HbA1c level in control rats was 3.9 ± 0.1% on day 1, and no significant change was observed in HbA1c level during the experimental period (Table 3a). HbA1c was significantly increased in the diabetic group compared to control group (5.97 ± 0.12% *vs.* 3.95 ± 0.10%; F(4,32) = 87.65, *p* < 0.001). At the end of the experimental period, there was a dramatic increase in HbA1c level in the diabetic group compared to their initial day value (11.92 ± 0.59% *vs*. 5.97 ± 0.12%, *p* < 0.001). After 42 days of extract treatment, HbA1c level was significantly (*p <* 0.001) decreased in AM-treated group (8.20 ± 0.18%) compared to the untreated diabetic group (11.92 ± 0.59%). Aminoguanidine, a known glycation inhibitor, also reduced HbA1c levels significantly (9.05 ± 0.35%, *p* < 0.001).

**Table 3:** Chronic effect of *A. marmelos* extract on HbA1c (A) and AGEs (B) in the diabetic rats

A.		
	HbA1c (%)	
Groups	Day 1	Day 43
Control (*n* = 11)	3.95 ± 0.10	4.06 ± 0.13
Db (*n* = 8)	5.97 ± 0.12	11.92 ± 0.59
AM (*n* = 6)	5.88 ± 0.07	8.20 ± 0.18***
AG (*n* = 6)	6.01 ± 0.05	9.05 ± 0.35***
B.		
	AGEs (mg/ml)	
Groups	Day 43	
Control (*n* = 11)	0.55 ± 0.06	
Db (*n* = 8)	1.18 ± 0.19	
AM (*n* = 6)	0.66 ± 0.05*	
AG (*n* = 6)	0.58 ± 0.10**	

All values were expressed as mean ± SEM. Control, non-diabetic control rats; Db, untreated diabetic rats; AM, *A. marmelos-*treated rats*;* AG, aminoguanidine-treated rats. *n* = number of rats in each group. *p < 0.05, **p < 0.01, ***p < 0.001, compared with Db values (one-way ANOVA with Dunnett’s multicomparison test)

### Effect of chronic AM extract treatment on circulating AGEs level in experimental rats

In control rats, the AGEs level was 0.55 ± 0.06 mg/ml (Table 3b). In the untreated diabetic rats, AGEs level was significantly (F(4,32) = 21.460, *p <* 0.001) increased compared to the control rats (1.18 ± 0.19 mg/ml *vs.* 0.55 ± 0.06 mg/ml). Interestingly, after AM extract treatment, AGEs level was significantly (*p <* 0.002) reduced. Aminoguanidine also decreased AGEs level significantly (0.58 ± 0.10 mg/ml, *p <* 0.001).

### Correlation of AGEs with different biochemical parameters in experimental rats

**Fig. 3:**
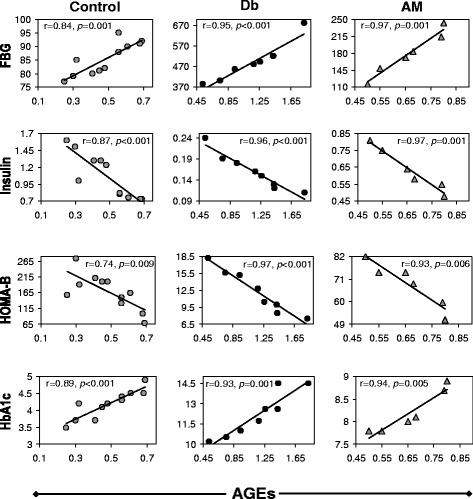
Correlation analysis of AGEs with fasting blood glucose, serum insulin, HOMA-B, and HbA1c done with one-way ANOVA with Dunnett’s multicomparison test

AGEs was positively correlated with fasting glucose and HbA1c in control, untreated diabetic and AM-treated groups, respectively (Fig. 3). A negative correlation was found between AGEs and serum insulin in all the groups. A negative correlation was also found between AGEs and β-cell function in all the groups.

### Effect of chronic AM extract treatment on histological changes of pancreas

**Fig. 4:**
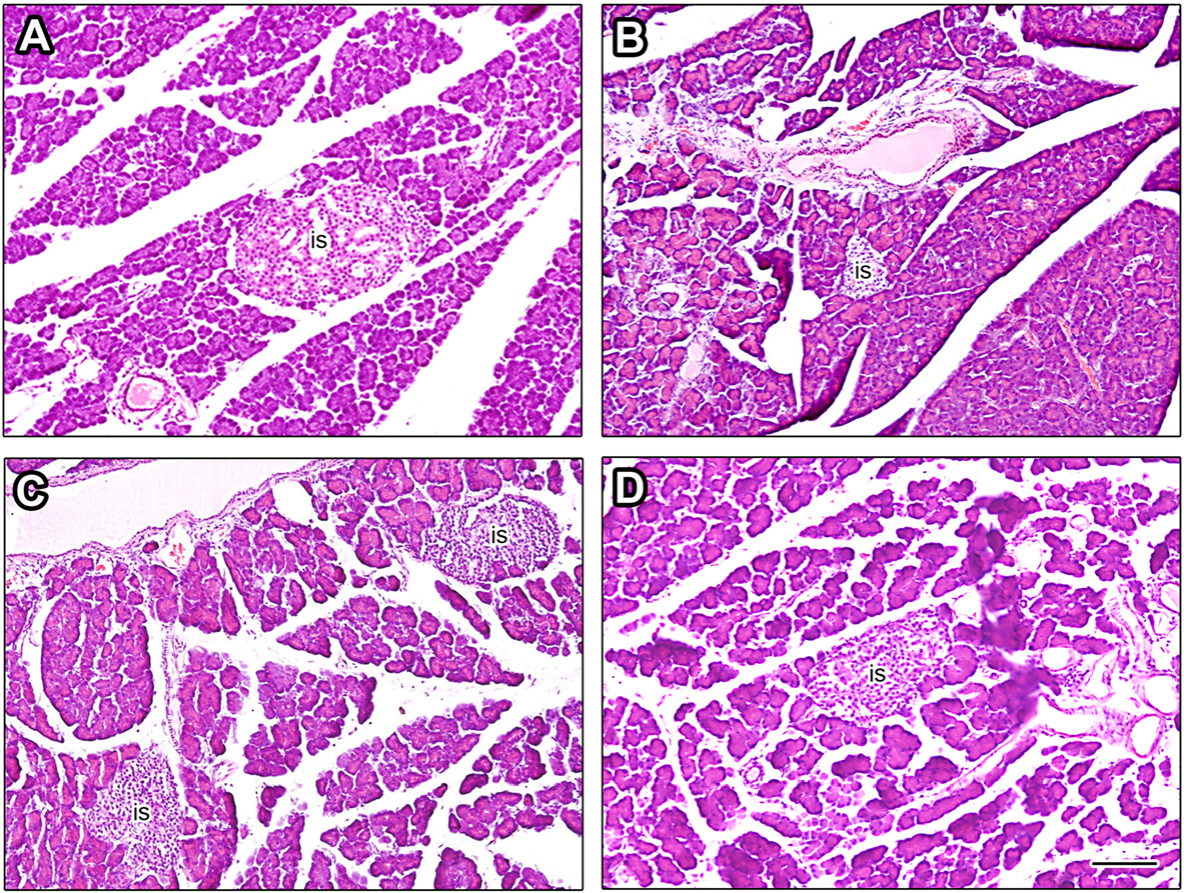
Histological comparison of the pancreatic tissue of the experimental rats. H&E staining of the pancreatic tissue from control **a** untreated diabetic **b** AM-treated (**c)** and GB-treated **d** diabetic rats. The islet shows reduced size in untreated diabetic rat as compared to control and treated rats. The representative images are shown from different pancreatic tissue sections from each group. AM, *A. marmelos*; GB, glibenclamide; is, islet. Scale bar in D = 100 μM

The H&E-stained pancreatic tissue section images from experimental groups are shown in Fig. 4. The H&E staining of pancreatic sections revealed that in control rats, the islets of Langerhans were abundantly distributed having moderate to large size with well-formed oval to round shape (Fig. 4a). In contrast, the pancreatic islets of the untreated diabetic rats were less distributed and almost all of the islets appeared to have reduced size with irregular shape, neither round nor oval (Fig. 4b). When the diabetic rats were treated with AM extract, the moderate distribution of pancreatic islets was observed and the islets appeared to have well-formed medium size with oval shape (Fig. 4c). AM extract was found to improve the pancreatic islets size but the extracts could not bring those to normal as seen in control rats. The standard drug Gb also improved the islets size (Fig. 4d).

### Effect of chronic AM extract treatment on pancreatic β-cells morphology

**Fig. 5:**
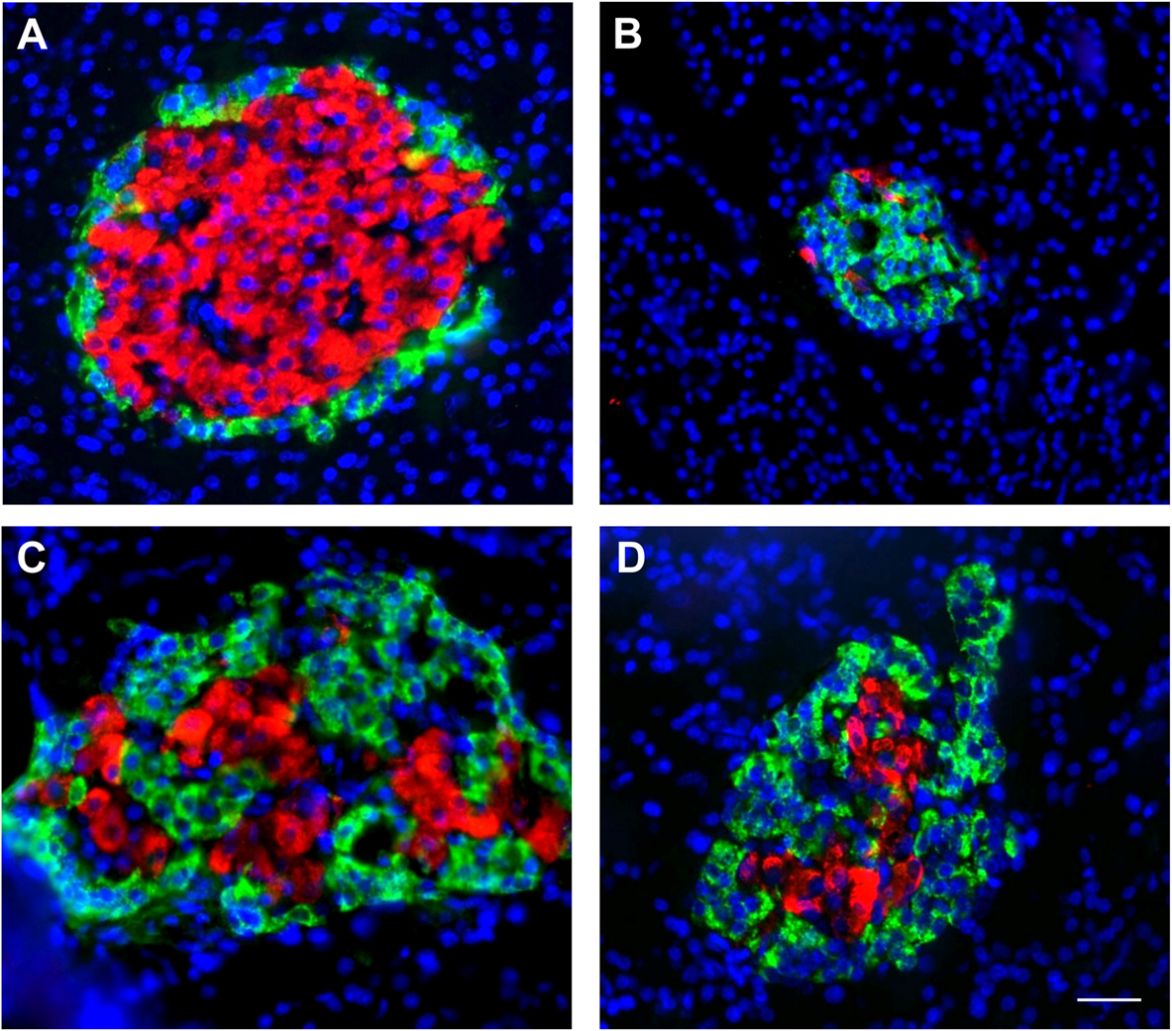
Immunohistochemical comparison of the pancreatic β- and α-cells of the experimental rats. Insulin (*red*) and glucagon (*green*) immunostaining in the pancreatic tissue from control **a** untreated diabetic **b** AM-treated (**c)**, and GB-treated **d** diabetic rats. Nuclei are stained with DAPI (*blue*). The representative images are shown from different pancreatic tissue sections from each group. AM, *A. marmelos*; GB, glibenclamide. Scale bar in D = 100 μM

The immunohistochemical images of insulin-expressing β-cells and glucagon-expressing α-cells along with the nuclei from the selected experimental groups are shown in Fig. 5. The data revealed that insulin-expressing cells were present in all these groups to different extent. In control rats, the distribution pattern of β-cells appeared normal showing abundant β-cells in the central position of islets (Fig. 5a). In contrast, the β-cells were reduced drastically in the untreated diabetic group (Fig. 5b). AM extract improved the insulin-expressing β-cells as compared to the untreated diabetic rats. When the diabetic rats were treated with AM extract, significant improvement was found in β-cells (Fig. 5c). The standard drug Gb also improved the β-cells (Fig. 5d).

In control rat islets, the α-cells were found at periphery of the β-cells. The relative distribution of β-cells was almost 70–80% and the distribution of α-cells was 15–20% (Fig. 5a). Interestingly, the glucagon-expressing α-cells were increased in the diabetic rats. There was an unusual mixed distribution pattern of β- and α-cells in the diabetic rats. In the diabetic rats, α-cells were found scattered within the central position of islets, whereas, the insulin expressing β-cells were found scattered at the periphery of islets (Fig. 5b). In AM-treated rats, improved distribution pattern of β- and α-cells was found (Fig. 5c). The Gb also improved the distribution pattern of β- and α-cells (Fig. 5d).

### Effect of chronic AM extract treatment on kidney histology

**Fig. 6:**
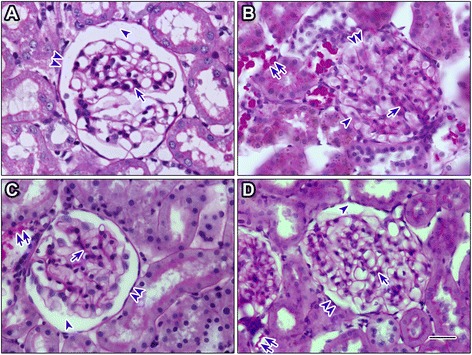
Histological comparison of the renal tissue of the experimental rats. PAS staining of the renal tissue from control **a** untreated diabetic **b** AM-treated (**c)** and AG-treated **d** rats. Glycogen deposition (*double arrows*), Bowman’s capsule (*double arrowheads*), Bowman’s space (*arrowhead*), mesangial area (*arrow*). The representative images are shown from different renal tissue sections from each group. AM, *A. marmelos*; AG, aminoguanidine. Scale bar in D = 100 μM

The images of PAS-stained kidney tissue sections were acquired from selected experimental groups by using light microscopy are shown in Fig. 6. The histopathological examination of renal tissue revealed that in control rats, there was no glycogen deposition (Fig. 6a). In contrast, the untreated diabetic group showed the high deposition of glycogen in their renal tubules (Fig. 6b). When the diabetic rats were treated with AM extract, the glycogen deposition was reduced compared to the untreated diabetic rats (Fig. 6c). In control rats, renal corpuscles showed the presence of intact capsular wall (Bowman’s capsule), normal capsular space (Bowman’s space) and mesangial area (Fig. 6a). But in case of untreated diabetic rats, the majority of renal corpuscles appeared to have distorted structure. The Bowman’s capsules were found distorted in certain places. There was considerable shrinkage in the Bowman’s space where in some places the Bowman’s capsule touched the glomerulus. Furthermore, the glomerulus mesangial area was found to be diffused and expanded (Fig. 6b). Upon the examination of AM-treated renal tissues, it was revealed that the Bowman’s capsule was almost intact and there was the restoration of Bowman’s space shrinkage as compared to the untreated diabetic group. The mesangial area diffusion and expansion was also reduced in AM-treated group compared to the untreated diabetic group (Fig. 6c). The treatment with AG also showed similar improvement in these regions (Fig. 6d).

### Effect of chronic AM extract treatment on AGEs and RAGE

**Fig. 7:**
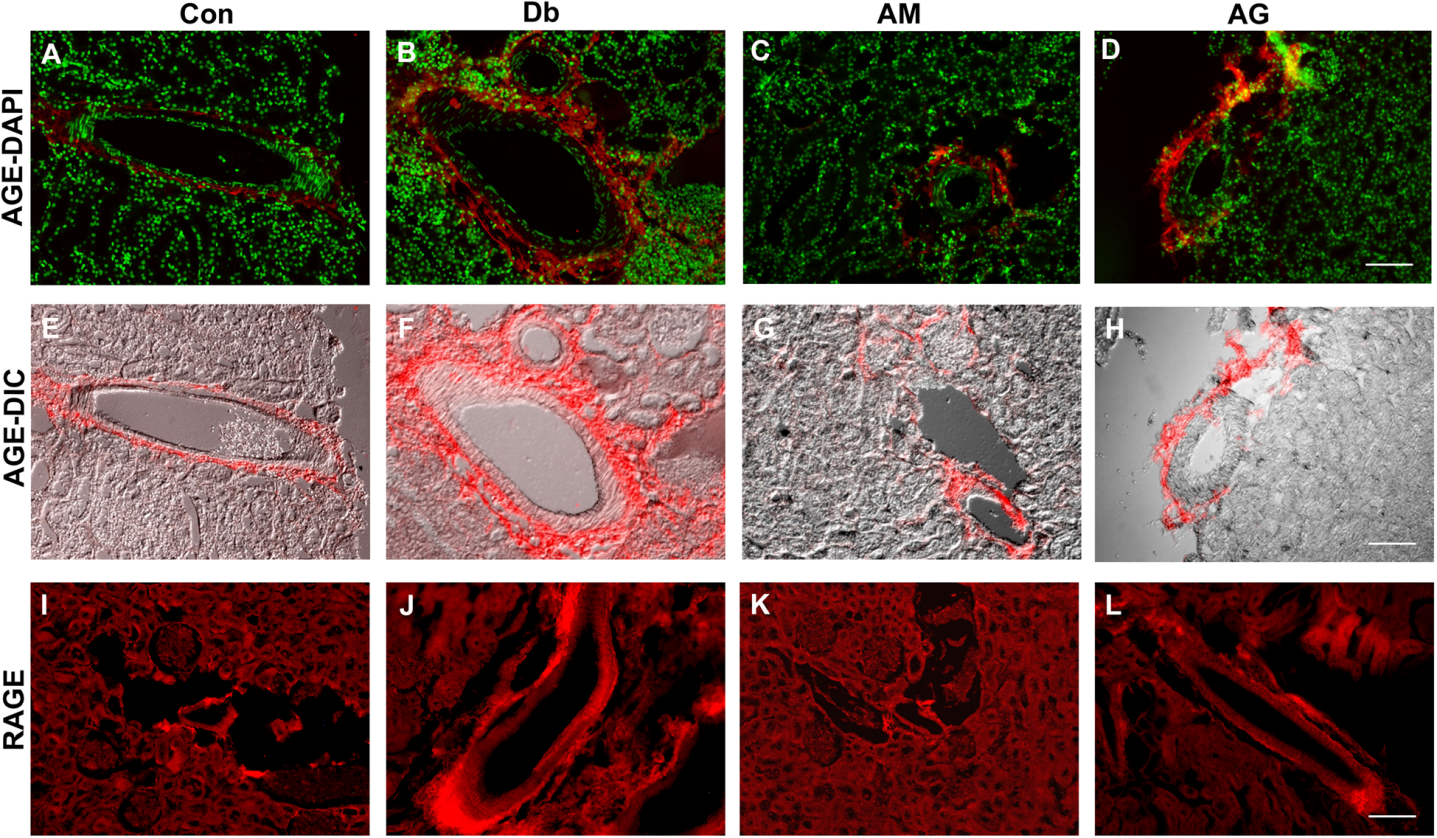
Immunohistochemical comparison of AGEs and RAGE in the renal artery of the experimental rats. Immunofluorescence of AGEs in the renal tissue from control (**a** and **e)** untreated diabetic (**b** and **f)** AM-treated (**c** and **g)**, and AG-treated (**d** and **h)** diabetic rats. An increased AGEs labeling around artery (*red*) appears in untreated diabetic rats compared to control and treated rats. Nuclei are stained with DAPI (*pseudocolored green*). The E-H are merged image of A-D and their respective differential interference contrast (DIC) microscopic image. Immunofluorescence of RAGE in the renal tissue from control **i** untreated diabetic **j** AM-treated (**k)**, and AG-treated **l** diabetic rats. The representative images are shown from different renal tissue sections from each group. AM, *A. marmelos*; AG, aminoguanidine. Scale bar = 100 μM

The distribution of AGEs in the kidney of different experimental groups was observed through immunohistochemical staining using fluorescence microscopy as shown in Fig. 7. In control rats, it was observed that there was very little to no immunofluorescence around the capsular wall (Bowman’s capsule) of the renal corpuscles and the tubular basement membrane (data not shown). The arteries were also appeared to show weak immunofluorescence (Fig. 7a). Interestingly, in the untreated diabetic rats the immunofluorescence labeling was increased considerably around the Bowman’s capsule as well as tubular basement membrane (data not shown). Very strong immunofluorescence was also observed around the arteries of the untreated diabetic rats (Fig. 7b). The reduction in fluorescence labeling around arteries, Bowman’s capsule and tubular basement membrane was found by AM extract (Fig. 7c). The AG also showed reduction of fluorescence labeling in these regions (Fig. 7d). The accumulation of RAGE is very similar to that of AGEs in diabetic rats (Fig. 6j) and RAGE accumulation was also prevented by AM treatment (Fig. 7k).

### Western blot analysis of AGEs, RAGE, and CML in kidney tissues in experimental rats

**Fig. 8:**
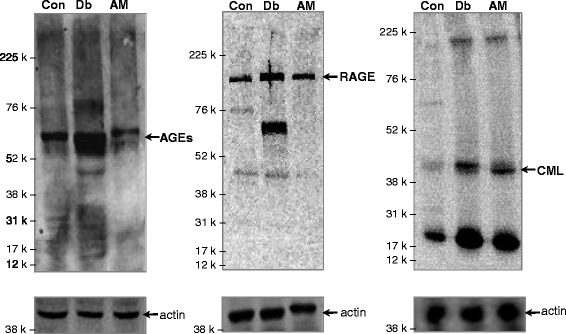
Western blots analyses of AGEs (*left panel*), RAGE (*middle panel*) and CML (*right panel*) in renal tissues of control, untreated diabetic and AM-treated diabetic rats. The whole kidney tissue lysates of 50 μg proteins were subjected to SDS-10% PAGE and Western blotting using anti-AGEs, anti-RAGE, and anti-CML antibody. Respective Western blots for β-actin (*lower panels*) used as a loading control was also shown. Rainbow high range molecular mass markers (RN-756E, GE Healthcare Life Sciences) are indicated on the left

Expression of AGEs, RAGE, and CML in kidney tissues of control, untreated diabetic and AM-treated diabetic rats is shown in Fig. 8. In control rats, a major band of AGEs of about 64 Kd was observed. In addition, three minor bands about 31 Kd, 24 Kd and 17 Kd were also found. The untreated diabetic kidney sample showed 64 Kd AGEs band with much more intense staining when compared to control. Along with 64 Kd AGEs, few thin bands were also observed above (80 Kd) and below (44 Kd, 31 Kd, 24 Kd and 17 Kd) the main thick band. In AM-treated rats, intensity of AGE major band was markedly decreased as compared to untreated one. In RAGE blot, a major band of RAGE was observed about 130 Kd in control rats. The untreated diabetic kidney sample showed two thick bands with intense staining, one band appeared at about 130 Kd and other appeared at around 64 Kd. In addition, one light band was also observed about 44 Kd. In AM-treated rat, intensity of 130 Kd band was markedly decreased compared to untreated one. In CML blot, the untreated diabetic kidney sample showed intense band of about 44 Kd when compared with control, where only background staining was seen. While in treated rat sample, intensity of band was decreased as compared to untreated one.

### Effect of chronic AM extract treatment on total antioxidant status

Changes of total antioxidant status (TAS) in each group of rats are shown in Table 4. Significant decrease of TAS was found in the untreated diabetic rats compared to the control rats (1.12 ± 0.09 mM *vs*. 1.89 ± 0.07 mM; F(3,27) = 11.40, *p* < 0.001). AM extract at 400 mg/kg for 42 days significantly increased the total antioxidant status (1.97 ± 0.12 mM, *p* < 0.001). A mild but significant increase of TAS (1.65 ± 0.08 mM, *p* = 0.013) was also found in Gb-treated rats.

**Table 4:** Chronic effect of *A. marmelos* extract on total antioxidant status in the diabetic rats

	Total antioxidant status (mM)
Groups	Day 43
Control (*n* = 11)	1.89 ± 0.07
Db (*n* = 8)	1.12 ± 0.17
AM (*n* = 6)	1.97 ± 0.12**
Gb (*n* = 6)	1.65 ± 0.08*

All values were expressed as mean ± SEM. Control, non-diabetic control rats; Db, untreated diabetic rats; AM, *A. marmelos-*treated rats*;* Gb, glibenclamide-treated rats. *n* = number of rats in each group. *p < 0.05, **p < 0.01, compared with Db values (one-way ANOVA with Dunnett’s multicomparison test)

### Effect of eugenol on fasting blood glucose in diabetic rats and glucose-stimulated insulin secretion in mice islets

**Fig. 9:**
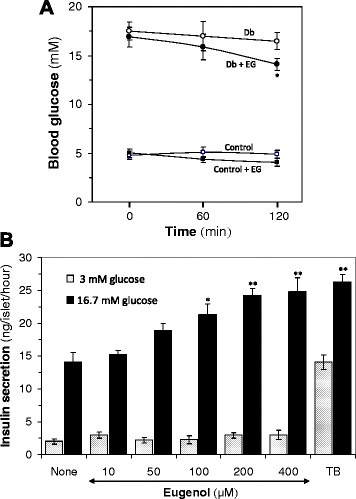
Acute effect of eugenol on fasting blood glucose levels in in vivo diabetic rats and glucose-stimulated insulin secretion in in vitro mice isolated islets. **a** EG was administered orally and blood glucose was measured at 0, 60 and 120 mins. Values are mean ± SD for five rats per group. Control, nondiabetic control rats; Control + EG, control rats treated with EG of 50 mg/kg; Db, diabetic control rats; Db + EG, diabetic rats treated with EG of 50 mg/kg. *P < 0.05 *vs.* 0-min value (paired *t*-test). **b** Groups of 3 size-matched mice isolated islets were incubated for one hour at 37 °C in KRB buffer, containing 3 mM or 16.7 mM glucose in the absence or presence of EG and secreted insulin was measured. Values are mean ± SEM from 3 independent experiments. ^†^ *p* < 0.05 compared with the value for 3 mM glucose alone; *p < 0.05, **p < 0.01 compared with the value for 16.7 mM glucose alone (one-way ANOVA with Dunnett’s multicomparison test). TB, tolbutamide (200 μM)

Acute effects of eugenol (EG) on blood glucose levels of control and diabetic rats are shown in Fig. 9a. After feeding EG to the control rats, little decrease in blood glucose level was found during the experimental period. In diabetic rats, EG showed reduction in blood glucose level that was not statistically significant at 60 min; however, the reduction was significant at 120 min (F(2,6) = 5.69, *p* < 0.028).

Insulin secretion activity of EG was performed in isolated mice islets. At basal glucose (3 mM), EG with 10–400 μM concentrations had little to no effects on insulin secretion (Fig. 9b). At stimulatory glucose (16.7 mM), 50 μM EG slightly enhanced insulin secretion which was not to the level of significance. Interestingly, 100 μM EG significantly (F(1,4) = 249.14, *p* = 0.042) increased insulin secretion (21.25 ± 1.71 ng/islet/h) compared with insulin secretion by 16.7 mM glucose alone (14.12 ± 1.41 ng/islet/h). No significant change was observed above the 100 μM dose of EG.

## Discussion

An accelerated formation of protein glycation occurs in diabetes due to higher blood glucose. The long-term glycation causes formation of AGEs, which contribute a key role to the pathogenesis of diabetic complications. Therefore, the suppression of AGEs formation is supposed to play an important role in the prevention and/or delay of diabetic complications. In this context, very recently, our group has undertaken a long-term program to assess antiglycation activity of commonly used dietary agents in Pakistan. The present work is the first to investigate AM fruit effects in STZ-induced diabetic rats.

Three different doses of AM extract (200, 400 and 600 mg/kg) along with Gb, were tested for OGTT to compare the effective dose and efficacy of AM A dose of 400 mg/kg produced significant reduction of blood glucose levels at 60, 120 and 180 mins and no appreciable reduction was observed on further increase of the dose (Fig. 1). Therefore, 400 mg/kg dose was chosen as the effective dose for chronic effects of the extract. The elevated levels of blood glucose were decreased significantly in AM-treated rats (Fig. 2). AM has also been reported for its anti-diabetic activity in diabetic animals [13, 19, 20]. So, our data further support the anti-diabetic activity of AM.

Serum insulin was dramatically decreased in our diabetic rats. Changes in serum insulin level in diabetes reflect abnormalities in β-cell function or insulin action. The decreased β-cell function (Table 1) or size of islets (Figs. 4– 5) is a clear indication that our diabetic rats have characteristics of β-cell dysfunction. The increased serum insulin by AM was comparable to that of Gb which suggested that this reduction of blood glucose may be due to the enhanced insulin secretion from the pancreatic β-cells. AM improved the β-cell function (Table 1), β-cell morphology (Fig. 5) and decreased glucose tolerance (Fig. 1) which suggest its effects on possible glucose-stimulated insulin secretion. Furthermore, compounds isolated from AM, including ellagic acid, ferulic acid, quercetin, rutin, eugenol stimulate glucose-induced insulin secretion in vitro [21–23] further suggest that reduction of blood glucose is most probably due to enhanced insulin secretion. Interestingly, we found that β-cells were increased with the treatments of AM in the diabetic rats (Fig. 5). This may be due to partial improvement of number of β-cells, and their activation, regeneration, revitalization or possibly due to inhibition of β-cell apoptosis [24–27]. Thus the immunohistochemical findings are in good agreement with our biochemical data as serum insulin level was significantly increased in diabetic rats treated with AM (Fig. 5 and Table 1).

It has been reported that AM fruit extract decreases HbA1c in diabetic rats [13]. Consistent to this observation, our results also showed significant decrease of HbA1c in diabetic rats by AM treatment. The decreased HbA1c level is most likely due to the reduction of blood glucose as we found a positive correlation between HbA1c and blood glucose level (Fig. 5). Other possibility is that, the compound(s) present in the AM extract may bind to Hb molecule and prevents HbA1c formation. Since AM showed significant antioxidant activity in vitro and in vivo (Table 4); therefore, the antiglycation effect of AM extract due to its antioxidant activity can not be ruled out.

In diabetes, the high blood glucose causes the accelerated formation of AGEs that circulate in blood and/or accumulate in certain tissues, which is one of the major causes of diabetic complications [28, 29]. Therefore, the status of AGEs in blood and/or in tissues is an important predictor of diabetic complications. In our study, AM dramatically decreased the circulating AGEs in diabetic rats (Fig. 3). The decrease may be due to the reduction in blood glucose level by AM that ultimately slow down the formation of AGEs. Other possibility is that, the presence of compound(s) in AM extract may sequester protein(s) or glucose thereby inhibiting the formation of AGEs. The net effect of AM may be due to the additive/synergetic effect by both mechanisms.

As mentioned previously, in addition to circulation AGEs are also accumulated in various organs e.g. kidney, eye, skin and other vascular tissues. The kidney is one of the major targets of AGE-mediated damage as a consequence of diabetes. Therefore, we further studied the accumulation of AGEs in renal tissue to find out whether the treatment by AM has preventive and/or delaying effect on AGEs accumulation. Studies have shown that the AGEs accumulation increases in Bowman’s capsule, glomerular basement membrane, glomerular mesangium and tubular basement membrane with the duration and severity of diabetes [30, 31]. Consistent to these studies we have also found AGEs accumulation around Bowman’s capsule and tubular basement membrane, and particularly around artery wall in diabetic rats (Fig. 7b). This may be due to hyperglycemia, which causes the endothelial cells to uptake more glucose and cause basement membrane thickening. AM decreased the accumulation of AGEs in the diabetic rats (Fig. 7c) possibly due to their protein glycation inhibitory as well as anti-diabetic activity.

Our immunoblotting data revealed that, more intense staining of AGEs, RAGE, and CML were observed **i**n the untreated diabetic rats compared to that of control; however, AM treatment significantly reduced the AGEs, RAGE, and CML in kidney tissues (Fig. 8). Along with AGEs major band of 64 Kd, few minor bands were found in untreated diabetic rats. The minor bands may be due to interaction of anti-AGEs antibody with some forms of AGEs and/or related proteins. In untreated diabetic sample, we observed two prominent, highly stained bands (130 Kd and 64 Kd) of RAGE. The 64 Kd thicker RAGE was only observed in diabetic kidney may be due to presence of some AGEs-modified proteins as RAGE ligands that may not exist under normal physiological condition. Other possibility is that monomeric RAGE appeared at 64 Kd while dimeric RAGE appeared at 130 Kd. Some minor bands in control as well as in diabetic kidney were also observed which may be due to interaction of anti-RAGE antibody with RAGE isomers. Band for CML was observed about 44 Kd in the experimental rats and interestingly in AGEs blot, there was a faint band about 44 Kd, which may be related to CML. In the RAGE blot, there was also a faint band about 44 Kd, which suggest possible interaction with CML.

On treatment with AM, the level of triglycerides and LDL-cholesterol was significantly decreased whereas HDL-cholesterol level significantly increased compared to that of untreated diabetic rats (Table 2). The decrease in triglycerides is most likely due to the insulin stimulatory effect of AM (Table 1), as insulin plays major role in the inhibition of lipolysis. The HDL-cholesterol level was increased due to improved insulin secretion by AM. The reduction in LDL-cholesterol is possibly because of the inhibition of glycosylation of LDL-cholesterol, as AM reduced the elevated blood glucose in the diabetic rats. Increased HDL and reduction in LDL suggest possible conversion of LDL to HDL and clearance of circulating lipids.

Administration of AM fruit extract prevents formation of advanced as well as early glycation in diabetic rats (Fig. 3 and Fig. 7), suggesting in vivo anti-glycation effect of AM fruit extract. Phytochemical studies have shown the presence of eugenol, ellagic acid, ferulic acid, and the flavonoid quercetin and its glycoside rutin (quercetin-3-rutinoside) in AM fruit extract [7–9]. Ellagic acid and ferulic acid has been reported for their insulin secretory activity [22, 23]. Very recently, Kittl et al. [32] reported that quercetin stimulates insulin secretion and reduces the viability of β-cells. Insulin secretion, pancreatic β-cell protection and anti-glycation effect of rutin was also reported [21, 33]. In the present study we also found that eugenol stimulates glucose-dependent insulin secretion from isolated mice islets (Fig. 9). Recently, Li et al. [34] reported that quercetin inhibits AGEs formation by trapping methylglyoxal and glyoxal in vitro.

## Conclusion

The anti-glycation effect of *A. marmelos* fruit extract may be due to the presence of quercetin and rutin that has direct effect on the prevention of AGEs or may be due to the presence of ellagic acid, ferulic acid, and eugenol that has insulin secretory effects that indirectly prevents AGEs formation by controlling elevated glucose. It is also possible that these polyphenols work in an additive or synergic manner through multiple mechanisms. Further studies are needed to determine the active principle and mechanism of action of the AM fruit extract.

## Abbreviations

AGEs: Advanced glycation end-products
RAGE: Receptor for AGEs
CML: N(6)-Carboxymethyllysine
TAS: Total antioxidant status
IGI: Insulinogenic index
AM: *Aegle marmelos*
AG: Aminoguanidine
Gb: Glibenclamide
